# Cellular Automata Framework for Classification of Cognitive Impairment from Clinical Dementia Rating

**DOI:** 10.1002/alz.087844

**Published:** 2025-01-09

**Authors:** Siva Manohar Reddy Kesu, Neelam Sinha, Hariharan Ramasangu

**Affiliations:** ^1^ M S Ramaiah University of Applied Sciences, Bangalore, karnataka India; ^2^ Centre for Brain Research (CBR), Indian Institute of Science, Bengaluru, Karnataka India; ^3^ Relecura, Bangalore, karnataka India

## Abstract

**Background:**

Clinical Dementia Rating (CDR) and its evaluation have been important nowadays as its prevalence in older ages after 60 years. Early identification of dementia can help the world to take preventive measures as most of them are treatable. The cellular Automata (CA) framework is a powerful tool in analyzing brain dynamics and modeling the prognosis of Alzheimer’s disease.

**Method:**

The proposed algorithm uses the CA framework to construct features for the classifier for the classification of classes in the dataset. A subject is assigned to a CA cell grid based on its feature values as rows and the specified number of cells in each row. When a CA grid receives a feature from a subject, the feature values are distributed among the cells using a transfer function. The distribution of featured values in the CA grid from the initialized cell to the neighboring cells in the row with a diffusion rate of 20%. Hence, redistributed CA images have been obtained for all the subjects. Deep learning architecture constituted with 4 layered Conv2d has been modeled for the classification of the CA images to classify low, moderate, and severe cognitive impairment.

**Result:**

CDR from the ADNI dataset comprising 1948 subjects has been preprocessed for the six features and three classes (i.e., Low, moderate, and severe cognitive impairment) with 70% train sets and 30% test sets. A balanced dataset of 89 subjects for moderate and severe cognitive impairment has given the classification accuracy of 96%. A balanced dataset of 363 subjects for low and moderate cognitive impairment has given the classification accuracy of 95%.

**Conclusion:**

A CA framework for the classification of cognitive impairment has been achieved with good accuracy. The implementation of the CA approach and its runtime performance has an advantage over the well‐known algorithms by giving a good pathway in contributing to the classification problems.

